# Author Correction: Solid-phase hetero epitaxial growth of α-phase formamidinium perovskite

**DOI:** 10.1038/s41467-020-19846-y

**Published:** 2020-11-12

**Authors:** Jin-Wook Lee, Shaun Tan, Tae-Hee Han, Rui Wang, Lizhi Zhang, Changwon Park, Mina Yoon, Chungseok Choi, Mingjie Xu, Michael E. Liao, Sung-Joon Lee, Selbi Nuryyeva, Chenhui Zhu, Kenny Huynh, Mark S. Goorsky, Yu Huang, Xiaoqing Pan, Yang Yang

**Affiliations:** 1 Department of Materials Science and Engineering, California NanoSystems Institute, University of California, Los Angeles, CA 90095 USA; 2 Department of Nanoengineering, SKKU Advanced Institute of Nanotechnology (SAINT), Sungkyunkwan University, Suwon, 16419 Republic of Korea; 3 Division of Materials Science and Engineering, Hanyang University, Seoul, 04763 Republic of Korea; 4 Department of Physics and Astronomy, University of Tennessee, Knoxville, TN 37996 USA; 5 Center for Nanophase Materials Sciences, Oak Ridge National Laboratory, Oak Ridge, TN 37831 USA; 6 Department of Materials Science and Engineering, Irvine Materials Research Institute, University of California, Irvine, CA 92697 USA; 7 Advanced Light Source, Lawrence Berkeley National Laboratory, Berkeley, CA 94704 USA

**Keywords:** Electronic devices, Synthesis and processing, Organic-inorganic nanostructures

Correction to: *Nature Communications* 10.1038/s41467-020-19237-3, published online 2 November 2020.

The original version of this Article contained an error in the author affiliations.

Mina Yoon was incorrectly associated with Department of Physics and Astronomy, University of Tennessee, Knoxville, TN 37996, USA. The affiliation of Mina Yoon with Center for Nanophase Materials Sciences, Oak Ridge National Laboratory, Oak Ridge, TN, 37831 USA was inadvertently omitted.

This has now been corrected in both the PDF and HTML versions of the Article.

