# Supplementary material for: Solid-phase hetero epitaxial growth of α-phase formamidinium perovskite
Source: Nat Commun. 2020 Nov 2;11:5514. doi: 10.1038/s41467-020-19237-3 (PMC7608657; doi:10.1038/s41467-020-19237-3)
Supplement: Supplementary file 1 — Supplementary Information [file 41467_2020_19237_MOESM1_ESM.pdf]

Supplementary information

Solid-phase hetero epitaxial growth of α-phase formamidinium perovskite

Jin-Wook Lee*, Shaun Tan, Tae-Hee Han, Rui Wang, Lizhi Zhang, Changwon Park, Mina Yoon, Chungseok Choi, Mingjie Xu, Michael E. Liao, Sung-Joon Lee, Selbi Nuryyeva, Chenhui Zhu, Kenny Huynh[1], Mark S. Goorsky, Yu Huang, Xiaoqing Pan, and Yang Yang*

Correspondence to:

J.-W. L, email: jw.lee@skku.edu

Y.Y., email: yangy@ucla.edu

Supplementary Note 1| Difference between PEA_2PbI_4 and $FPEA_2PbI_4$.

In **Supplementary Fig. 1**, we performed density functional theory (DFT) calculations to investigate the charge distributions in the phenethylammonium (PEA) and 4-fluoro-phenethylammonium (FPEA) cations to gain insights on the properties of the layered perovskites incorporated with the cations. Due to the strong electronegativity of fluorine, a strong localized negative charge of -0.072e was observed on the fluorine in the FPEA cation, which is relatively higher than the positive charges being delocalized on the benzene ring in the PEA cation without the fluorine (0.042e and 0.035e). We speculate that this localized negative charge on the fluorine will disturb the assembly of the PbI_6-FPEA-FPEA-PbI_6 geometry when constructing the layered perovskite lattice due to the repulsive electrostatic force between the fluorines adjacent to each other in the two FPEA cations. Thus, the formation of the long-range ordered large crystals of layered $FPEA_2PbI_4$ perovskite will be less favorable than that for the layered PEA_2PbI_4 perovskite. In **Supplementary Fig. 2**, the X-ray diffraction (XRD) patterns of the PEA_2PbI_4 and $FPEA_2PbI_4$ films were measured to confirm our hypothesis. In fact, the overall peak intensity of the $FPEA_2PbI_4$ film was lower than the PEA_2PbI_4 film, and the full-width-half-maximums (FWHMs) for the peaks were relatively higher (0.06998 for the 002 orientation peak) than that of PEA_2PbI_4 (0.06280 for the 002 orientation peak), as shown in **Supplementary Fig. 2b**. Therefore, we speculate that the added $FPEA_2PbI_4$ in the $FAPbI_3$ precursor solution will aggregate less, and will be more dispersed in the film than PEA_2PbI_4. Therefore, a larger hetero-interfacial area is expected with the addition of $FPEA_2PbI_4$, as compared to the case with PEA_2PbI_4 (i.e. the area of the hetero-interface is larger for 3F than that for 3P).

Supplementary Note 2 | Fitting of isothermal transformation diagrams.

The *Johnson-Mehl-Avrami* (JMA) equation is a simplified model to relate the fraction of transformed phase, *f*, to the time elapsed, *t*, by the equation 1[1]:

$$f = 1 - \exp(-\frac{\pi}{3}Nv^3t^n) = 1 - \exp(-kt^n) \quad (1)$$

where $k = \frac{\pi}{3}Nv^3$ and *N* and *v* are the nucleation and growth rates, respectively. The exponent *n* relates to the growth dimensionality. For instance, *n* = 4 relates to 3 dimensions of growth of a spherical nuclei + 1 due to a constant nucleation rate[1,2]. For heterogenous nucleation (for instance, on grain boundaries) and for growth of thin films, *n*=3 is generally assumed[1,2].

The form of the JMA equation can be intuitively rationalized by considering a simplified derivation for nuclei growing as spheres as follows, the volume of a nuclei, *V*, with radius *r*, at time *t* = 0 is given by the equation 2:

$$V = \frac{4}{3}\pi r^3 = \frac{4}{3}\pi(vt)^3 \quad (2)$$

A nucleus which does not nucleate until time *τ* will have volume:

$$V' = \frac{4}{3}\pi v^3(t-\tau)^3 \quad (3)$$

The number of nuclei that forms in a time d*τ* will be *N*d*τ*. Assuming that the nuclei do not impinge on one other, the fraction of transformed phase will thus be given by:

$$f = \sum V' = \frac{4}{3}\pi N v^3 \int_0^t (t-\tau)^3 \, \mathrm{d}\tau = \frac{\pi}{3}Nv^3t^4 \quad (4)$$

This expression is valid for spherical nuclei of the case where *f* << 1 where no grain impingement occurs. Impingement decreases the phase transformation rate, such that the general equation for *f* taking into account the effect of impingement is of the form:

$$f = 1 - \exp(-\frac{\pi}{3}Nv^3t^4) \quad (5)$$

The general equation simplifies to the previous equation at short times since $1 - \exp(-z) \cong z$ when *z* << 1. Additionally, as $t \to \infty$, $f \to 1$, which is expected.

Supplementary Note 3 | Williamson-Hall analysis.

It is known that size-induced and strain-induced peak broadening vary differently as a function of Bragg angle, θ, where:

$$\beta_{\text{size}} = \frac{\mathrm{K}\,\lambda}{L\cos\theta} \quad (6)$$

$$\beta_{\text{strain}} = 4\varepsilon\frac{\sin\theta}{\cos\theta} \quad (7)$$

where β_{size} and β_{strain} are size and strain broadening, respectively, K is a constant depending on crystallite shape, λ is the x-ray wavelength, L is the crystallite size and ε is the lattice strain. For spherical crystals with cubic symmetry, K is taken to be 0.94.[2]

The Williamson-Hall method [3] assumes that the total broadening, β_{total}, given by the full width at half maximum (FWHM) of an individual peak, is a simple sum of the two effects, where:

$$\beta_{\text{total}} = \beta_{\text{size}} + \beta_{\text{strain}} = \frac{\mathrm{K}\,\lambda}{L\cos\theta} + 4\varepsilon\frac{\sin\theta}{\cos\theta} \quad (8)$$

Multiplying throughout by cos θ gives:

$$\beta_{\text{total}}\cos\theta = \frac{\mathrm{K}\,\lambda}{L} + 4\varepsilon\sin\theta \quad (9)$$

Therefore, a plot of $\beta_{\text{total}}\cos\theta$ against $\sin\theta$ produces a linear plot whereby the slope is proportional to the strain ε and intercept is inversely proportional to the crystallite size L. The X-ray diffraction patterns were fitted to Lorentzian distribution function. The fitted peak positions and full-width-half-maximums of the peaks are summarized in **Supplementary Table 2**. The corresponding plots in **Supplementary Fig. 10** is fitted to a linear curve, and the results are summarized in **Supplementary Table 3**.

Supplementary Note 4 | Capacitance-voltage characteristics of the LEDs.

The charge carrier behavior of the perovskite LEDs according to a given applied bias was estimated by impedance spectroscopy (**Supplementary Fig. 22**). The initial increase of the capacitance before the turn-on voltage (V_{th}~1.3 V) generally indicates majority charge carrier injection from the electrodes into the diode, and the injected majority carriers accumulate at one of the interfaces, increasing capacitance rapidly. When the voltage reaches the V_{th} of the LED, minority carrier injection from the counter electrode begins to decrease the capacitance accumulated at the emitting layer interface by electron-hole recombination. Therefore, the sharp increase and then decrease in capacitance close to V_{th} indicates effective charge carrier/ exciton blocking and balanced charge transport inside the perovskite emitting layer, resulting in enhanced radiative recombination. The LED with 3F as its emitting layer shows apparent peak capacitance at about 1.3 V, whereas the control device does not show effective charge blocking characteristics and the capacitance decreases before turn-on. This obvious difference in capacitance-voltage characteristics can be attributed to the type-I energy band alignment, and their effective charge blocking ability on the 3F perovskite crystal grains.

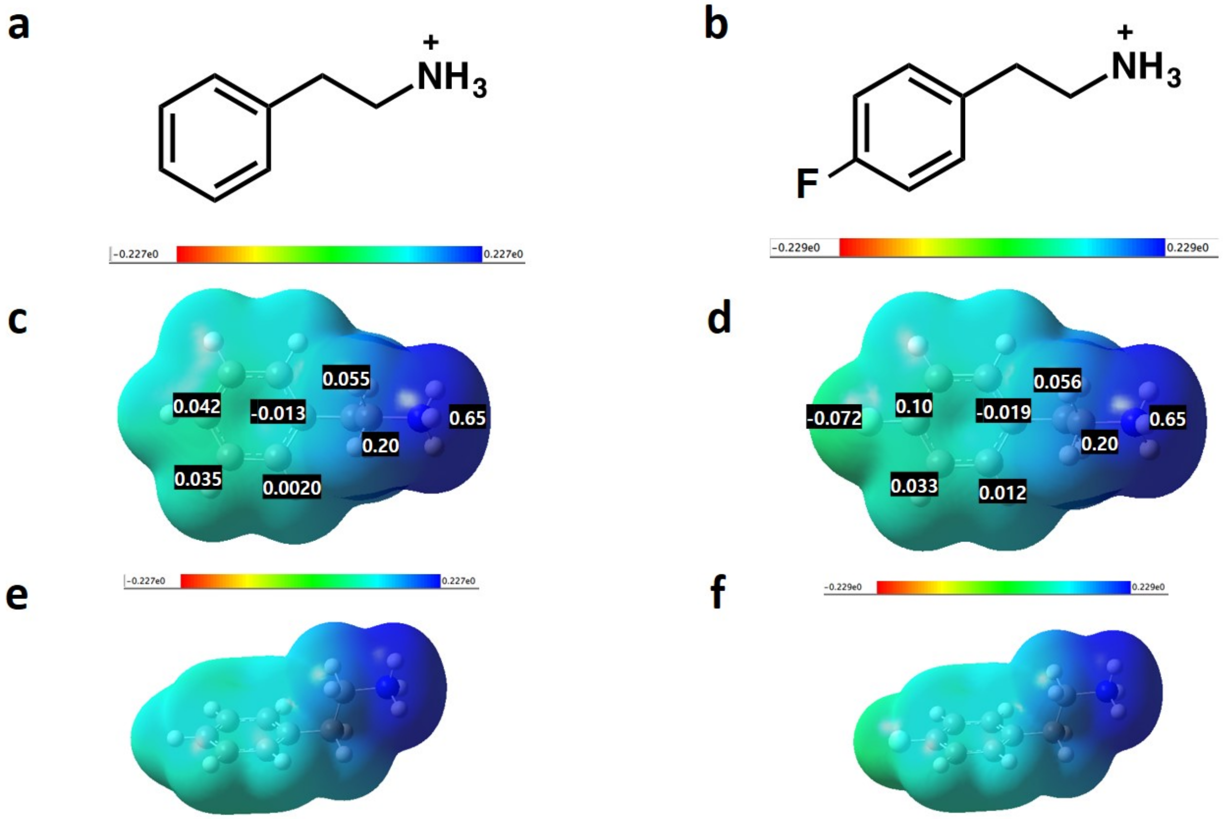

Supplementary Fig. 1| Charge distribution determined by density functional theory (DFT) calculation. Schematics showing molecular structure structure of **a**, phenethylammonium cation (PEA^+) and **b**, 4-fluoro-phenethylammonium cation ($FPEA^+$). Charge distribution of the **c**, **e**, PEA^+ and **d**, **f**, $FPEA^+$ molecules calculated by DFT calculation. **c**, **d**, top and **e**, **f**, cross sectional views

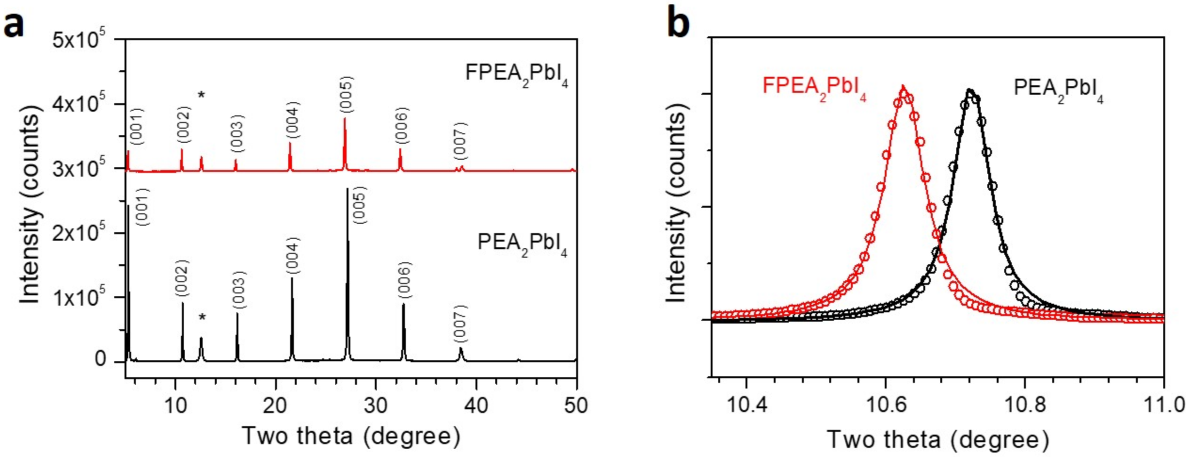

Supplementary Fig. 2 | X-ray diffraction (XRD) patterns of PEA_2PbI_4 and $FPEA_2PbI_4$ films. a, XRD patterns of PEA_2PbI_4 and $FPEA_2PbI_4$ films on a glass substrate. The peaks indexed by * is from (001) orientation of PbI_2. **b**, The normalized (002) orientation peaks where the open circles are measured data and solid lines are the fitted curves using a Lorentzian distribution function.

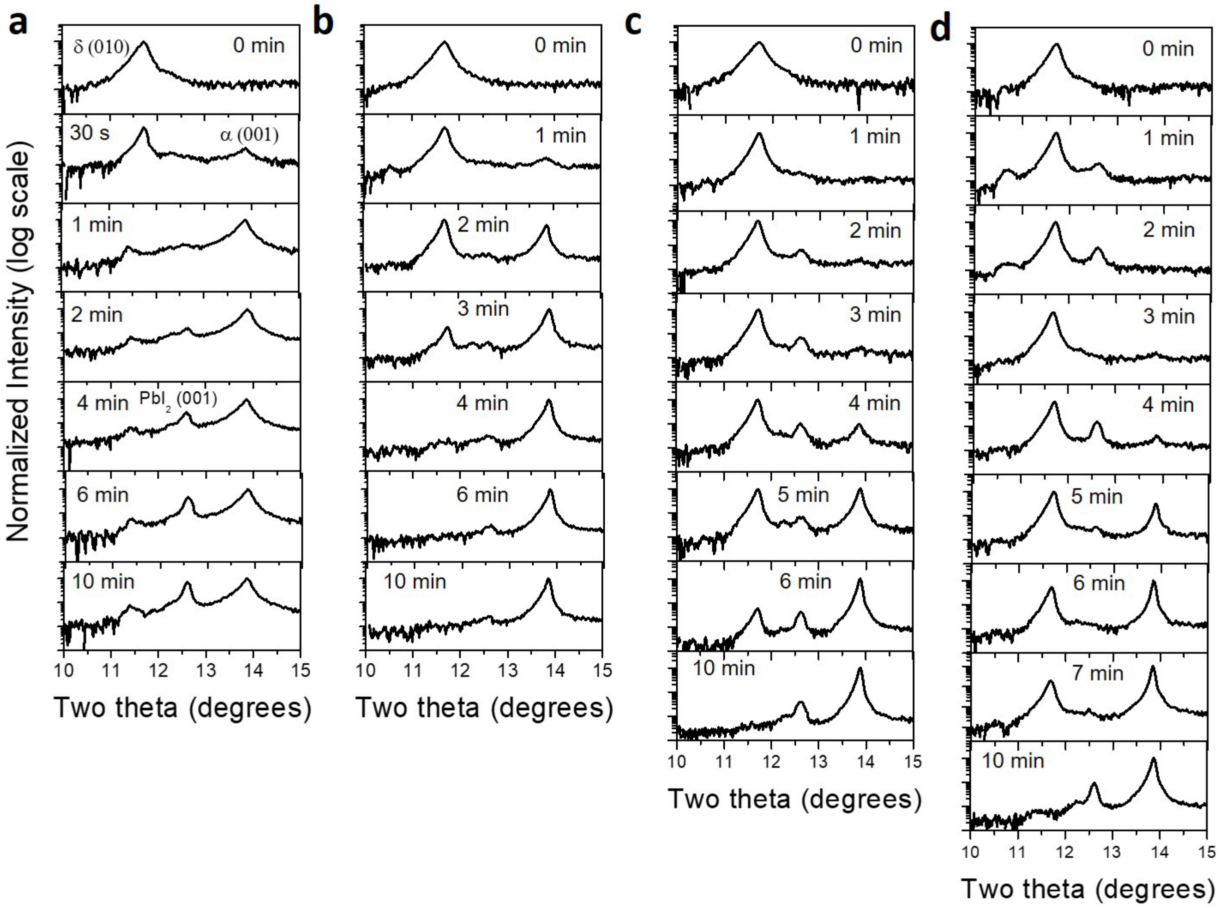

Supplementary Fig. 3 | X-ray diffraction (XRD) patterns of $FAPbI_3$ films with different compositions and annealing time at 150 °C. a, control: bare $FAPbI_3$, **b**, 1P: $FAPbI_3$ with 1.67 mol% PEA_2PbI_4, **c**, 3P: $FAPbI_3$ with 3.33 mol% PEA_2PbI_4, and **d**, 3F: $FAPbI_3$ with 3.33 mol% $FPEA_2PbI_4$.

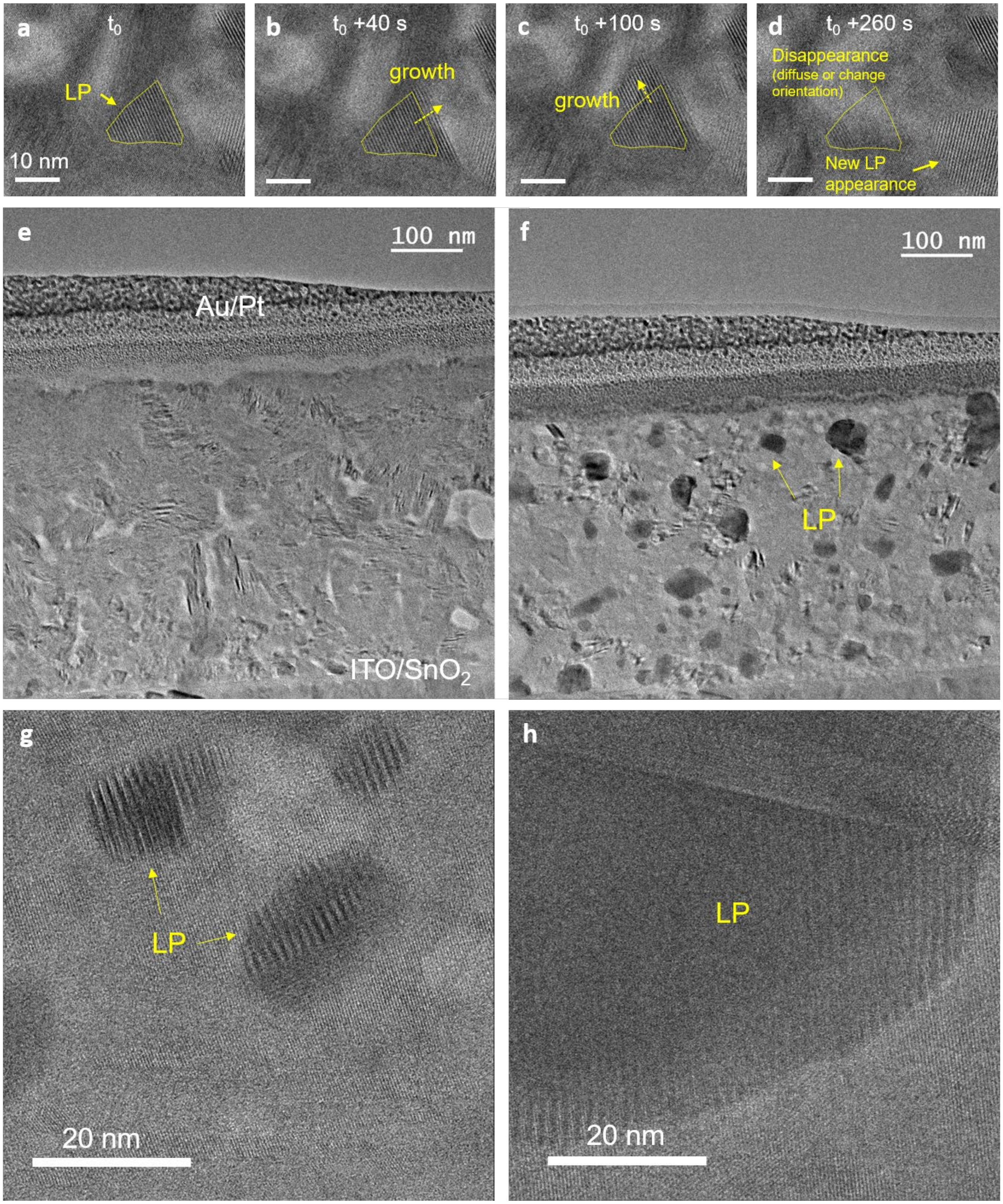

Supplementary Fig. 4 | a-d, In-situ cross-sectional transmission electron microscopy (TEM) images of the layered perovskite (LP) in the $FAPbI_3$ film incorporated with 3.33 mol% $FPEA_2PbI_4$. The film was heated for different times to induce the phase transition of $FAPbI_3$ from its hexagonal phase to cubic polymorph. Cross sectional TEM images of $FAPbI_3$ films with

3.33 mol% $FPEA_2PbI_4$ e, before and f, after the in-situ measurement to induce the phase transition. Thickness of the sample was around 50 nm. g,h, The magnified TEM images after the in-situ measurement, showing interface between LP phase and epitaxially grown $FAPbI_3$.

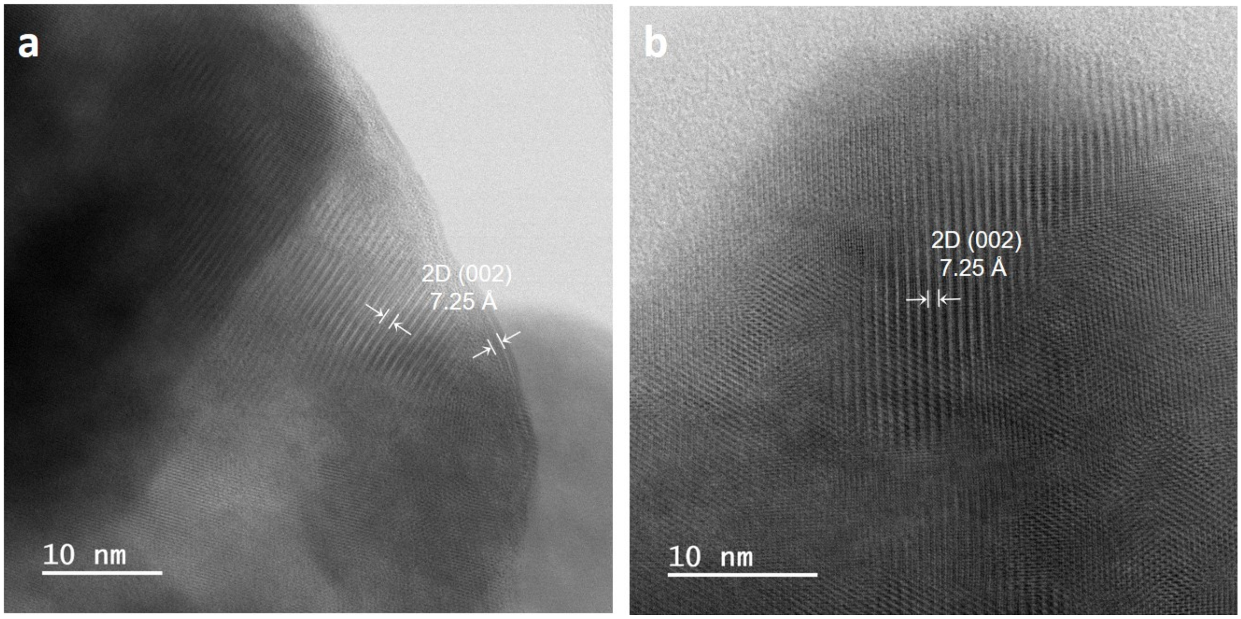

Supplementary Fig. 5 | Scanning transmission electron microscopy images of $FAPbI_3$ films with 3.33 mol% $FPEA_2PbI_4$, **a**, before phase transition **b**, after phase transition. The samples were prepared by scratching off the films from the substrate.

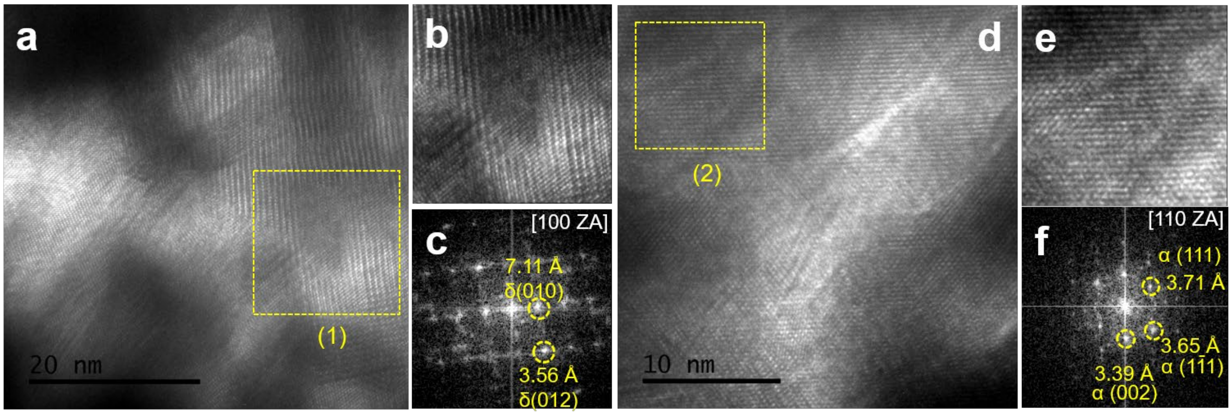

Supplementary Fig. 6 | Transmission electron microscopic (TEM) analysis $FAPbI_3$ films. TEM images of the bare hexagonal **a**, **b**, and cubic **d**, **e**, $FAPbI_3$ film. **b** and **e** shows the magnified image of region 1 and 2, respectively. **c** and **f** show FFT analysis of the region 1 and 2 respectively. The samples were prepared by scratching off the films from the substrate.

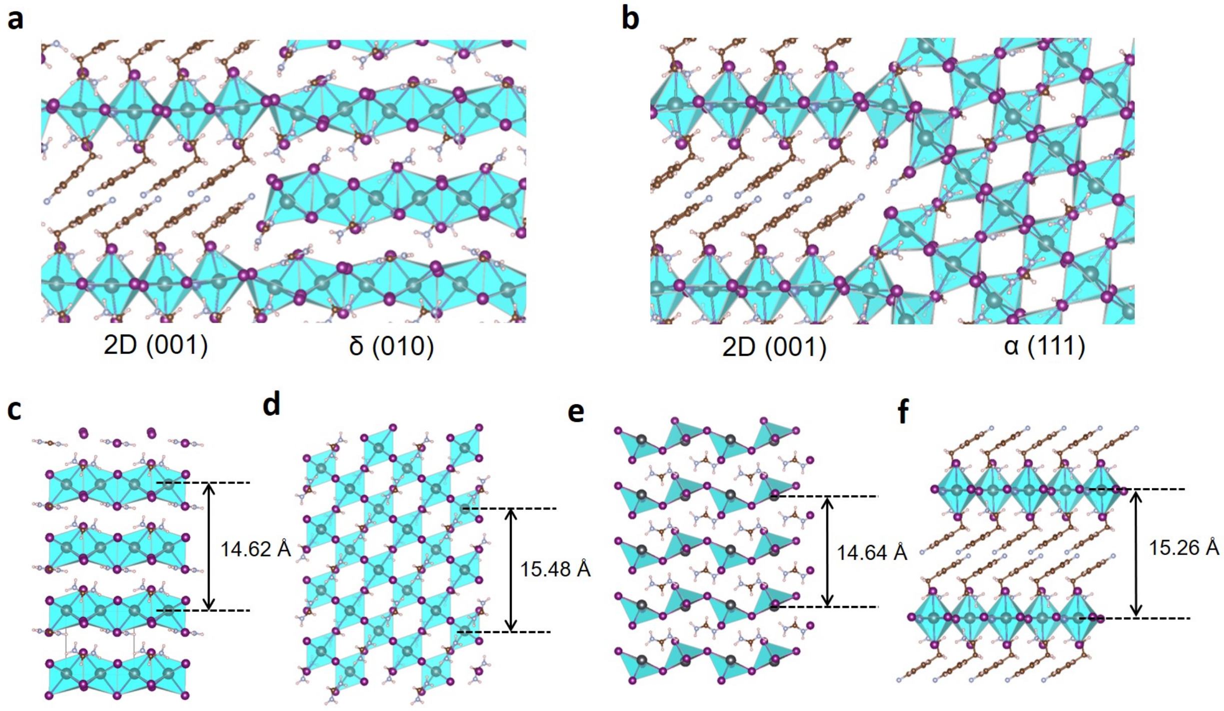

Supplementary Fig. 7| Schematics of perovskite crystal structure and their interfacial alignments. a, The interface between the hexagonal $FAPbI_3$ and $FPEA_2PbI_4$ crystals and **b**, the interface between the cubic $FAPbI_3$ and $FPEA_2PbI_4$ crystals determined by transmission electron microscopy studies. **c**-**f**, Density functional theory (DFT) optimized crystal configurations of $FAPbI_3$ and layered perovskite. δ-$FAPbI_3$ **c**, α-$FAPbI_3$ **d**, and transition state with the highest energy (saddle point, **e**) , and layered perovskite (**f**), where their interplanar distances are indicated in each figure. Purple spheres: iodine, black spheres: lead, brown spheres: carbon, white spheres: hydrogen

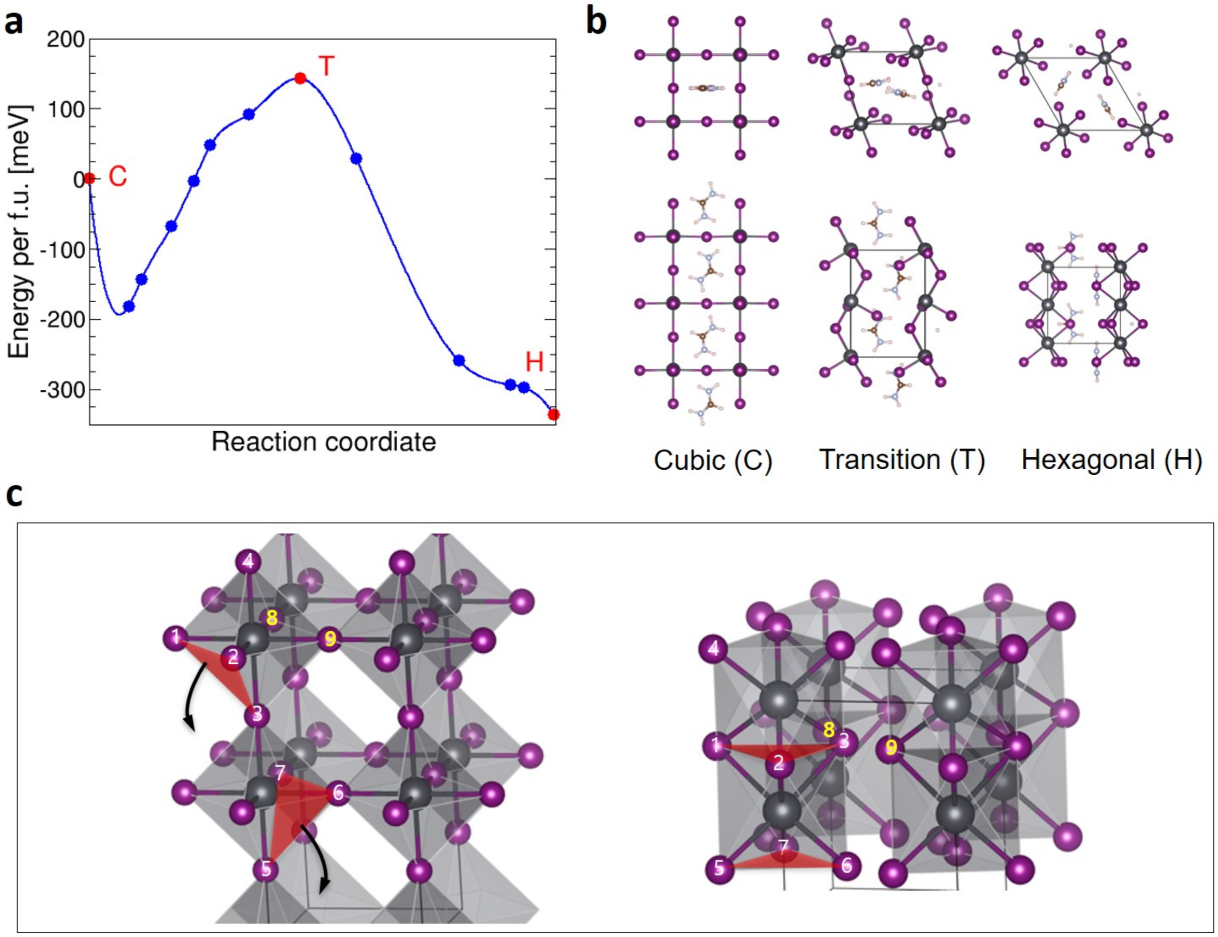

Supplementary Fig. 8 | Phase conversion energetics without strain a, Energy landscape of the phase conversion of $FAPbI_3$. **b**, Atomic configurations of the cubic, hexagonal and transition state phases. **c**, Crystal models showing the phase conversion pathway where the atoms numbered to track their redistribution due to the phase conversion. Purple spheres: iodine, black spheres: lead, brown spheres: carbon, white spheres: hydrogen

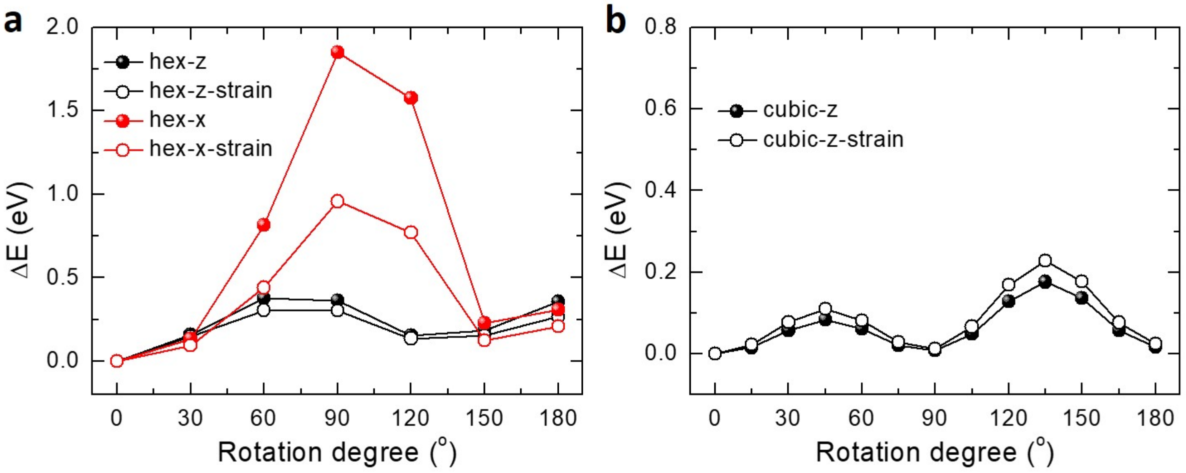

Supplementary Fig. 9 | Calculated energy landscape for ratational motion of the FA^+ molecule. **a**, The energy landscape of the rotational motion of FA^+ in hexagonal phase without and with strain. Two different orientation was calculated (z and x axis). **b**, The energy landscape of the rotational motion of FA^+ in cubic phase without and with strain.

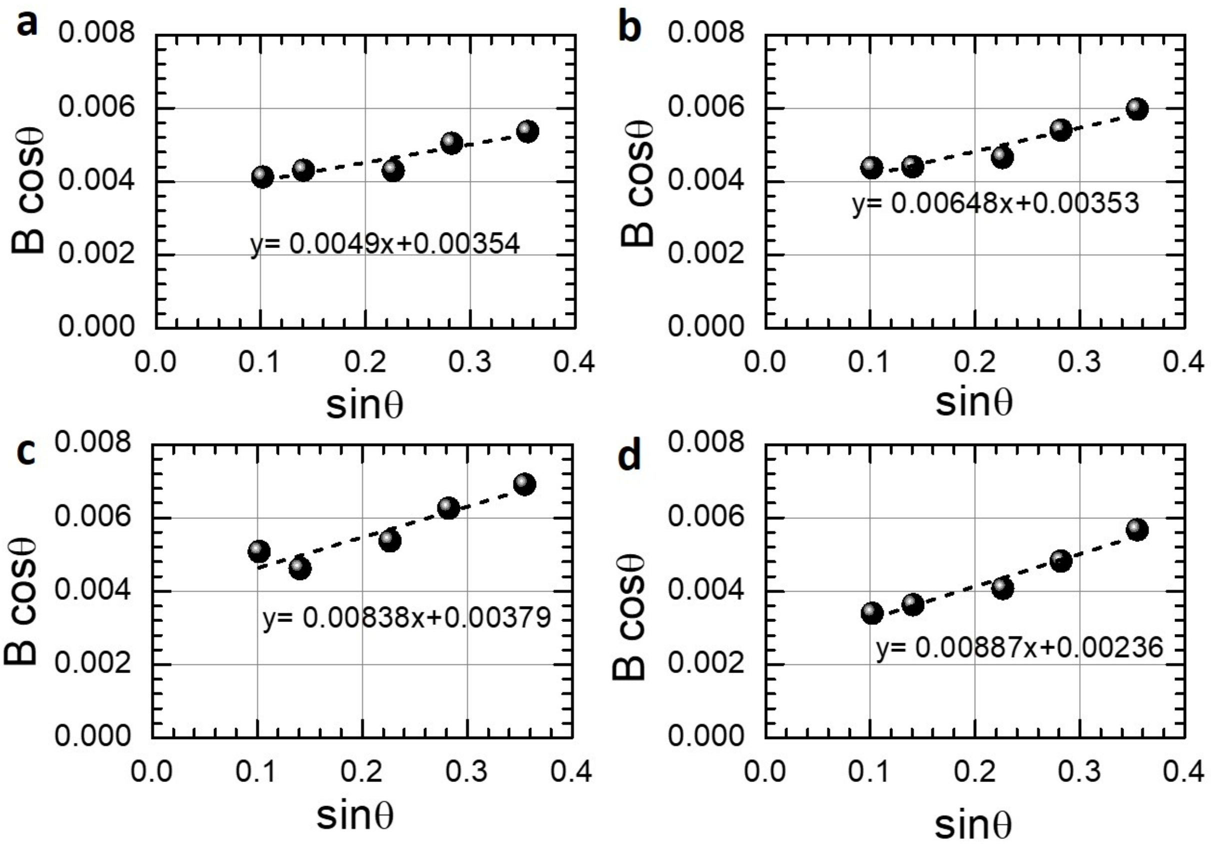

Supplementary Fig. 10 | Willamson-Hall analysis of hexagonal $FAPbI_3$ films with different composition. **a**, control: bare $FAPbI_3$, **b**, 1P: $FAPbI_3$ with 1.67 mol% PEA_2PbI_4, **c**, 3P: $FAPbI_3$ with 3.33 mol% PEA_2PbI_4, **d**, 3F: $FAPbI_3$ with 3.33 mol% $FPEA_2PbI_4$. The filled curcles are measured data and dashed lines are fitted lines.

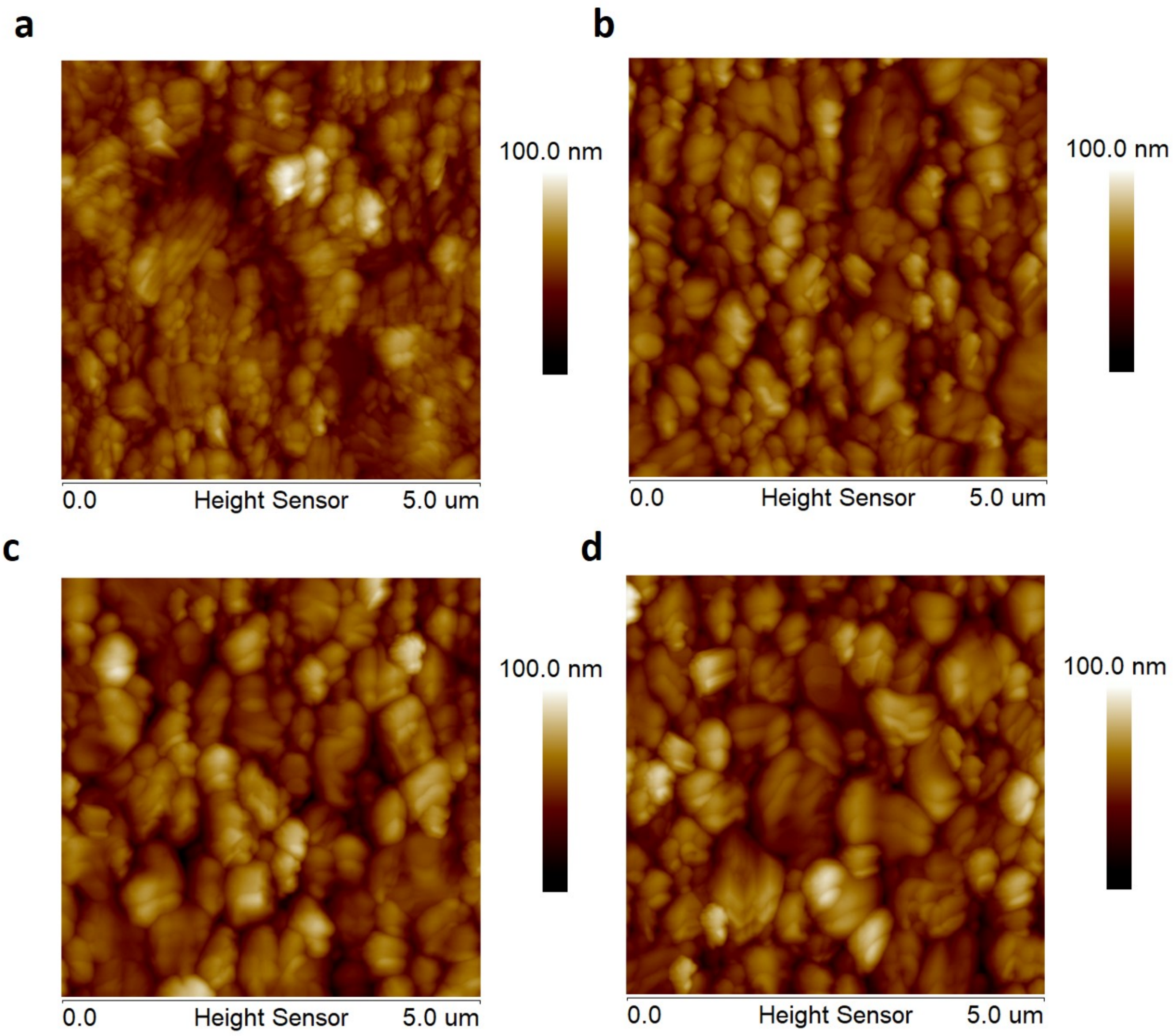

Supplementary Fig. 11 | Atomic force microscopic (AFM) images of $FAPbI_3$ films. a, control: bare $FAPbI_3$, **b**, 1P: $FAPbI_3$ with 1.67 mol% PEA_2PbI_4, **c**, 3P: $FAPbI_3$ with 3.33 mol% PEA_2PbI_4, **d**, 3F: $FAPbI_3$ with 3.33 mol% $FPEA_2PbI_4$.

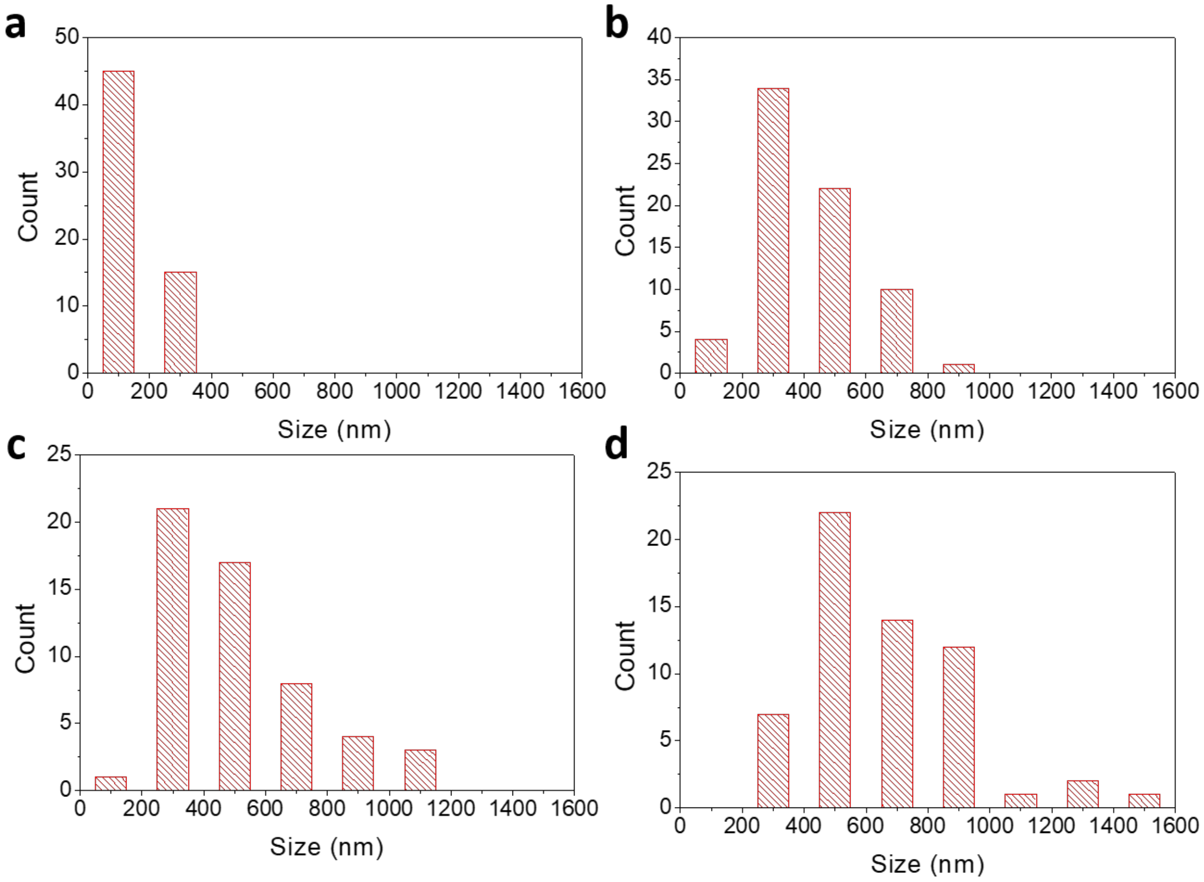

Supplementary Fig. 12 | Grain size distribution extracted from the atomic force microscopy images. a, control: bare $FAPbI_3$, **b**, 1P: $FAPbI_3$ with 1.67 mol% PEA_2PbI_4, **c**, 3P: $FAPbI_3$ with 3.33 mol% PEA_2PbI_4, **d**, 3F: $FAPbI_3$ with 3.33 mol% $FPEA_2PbI_4$.

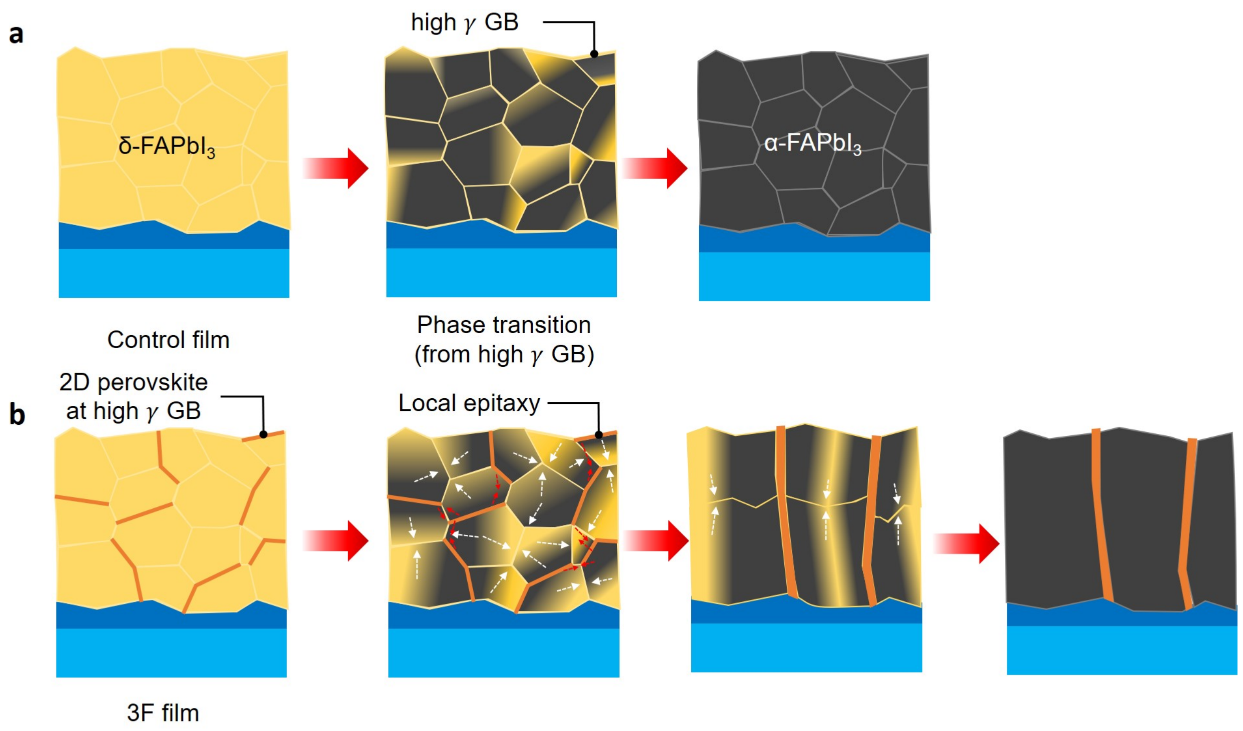

Supplementary Fig. 13 | Proposed mechanism for the bulk heteroepitaxy process. a, Phase conversion process of the bare $FAPbI_3$ film. **b**, Phase conversion process of $FAPbI_3$ with added layered perovskite. GB: grain boundary, $\boldsymbol{\gamma}$: surface energy

5

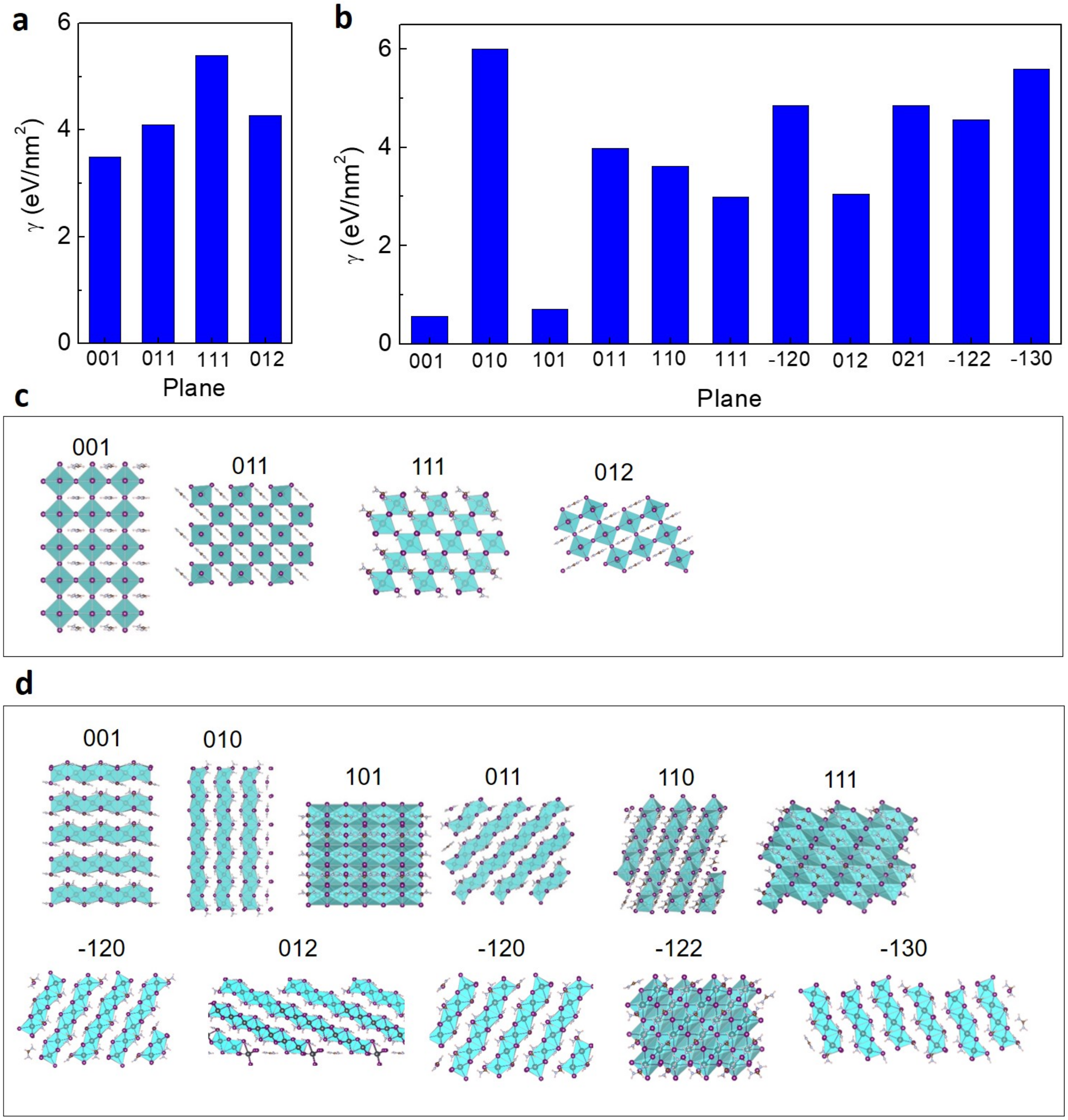

Supplementary Fig. 14 | Calculated surface energies for **a**, α-$FAPbI_3$ and **b**, δ-$FAPbI_3$ **c, d**, Crystal configurations used for the calculations; **c**, α-$FAPbI_3$, **d**, δ-$FAPbI_3$.

5

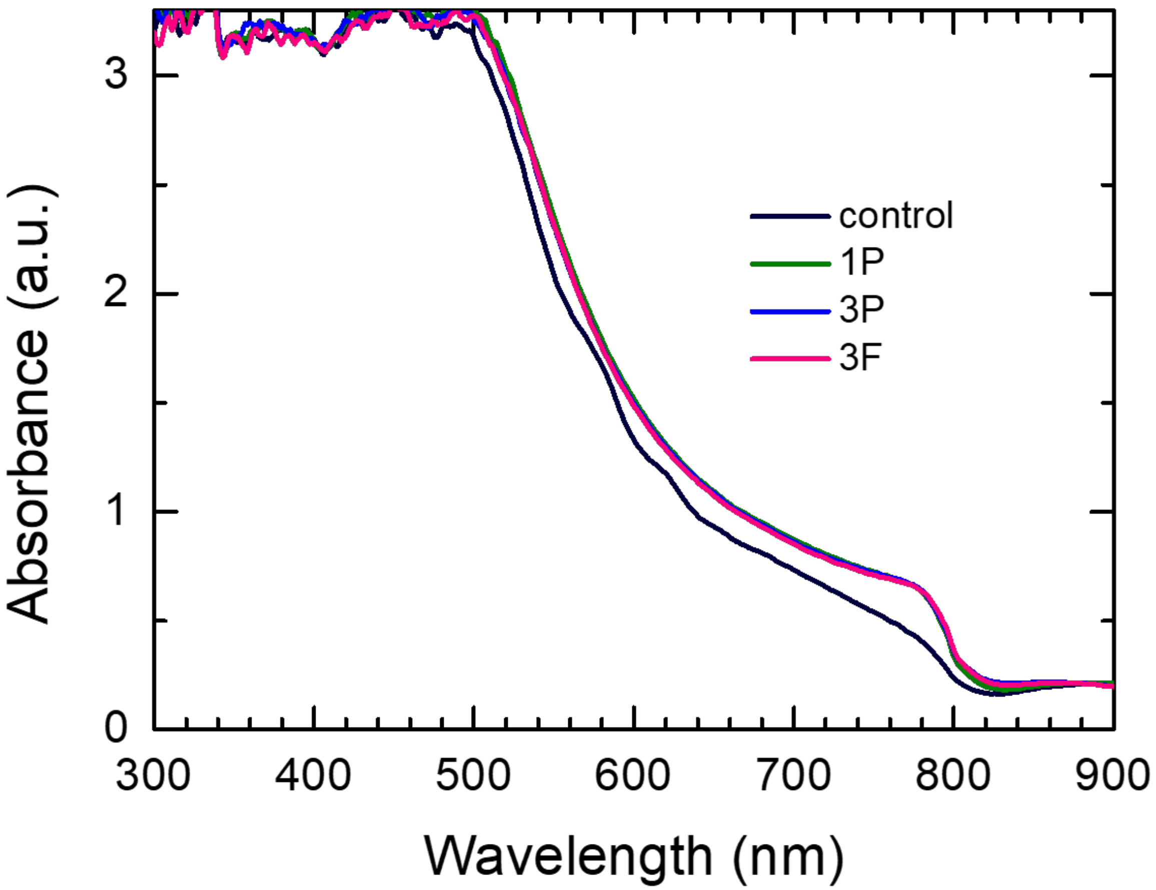

Supplementary Fig. 15 | Absorption spectra of $FAPbI_3$ films. Control: bare $FAPbI_3$, 1P: $FAPbI_3$ with 1.67 mol% PEA_2PbI_4, 3P: $FAPbI_3$ with 3.33 mol% PEA_2PbI_4, 3F: $FAPbI_3$ with 3.33 mol% $FPEA_2PbI_4$.

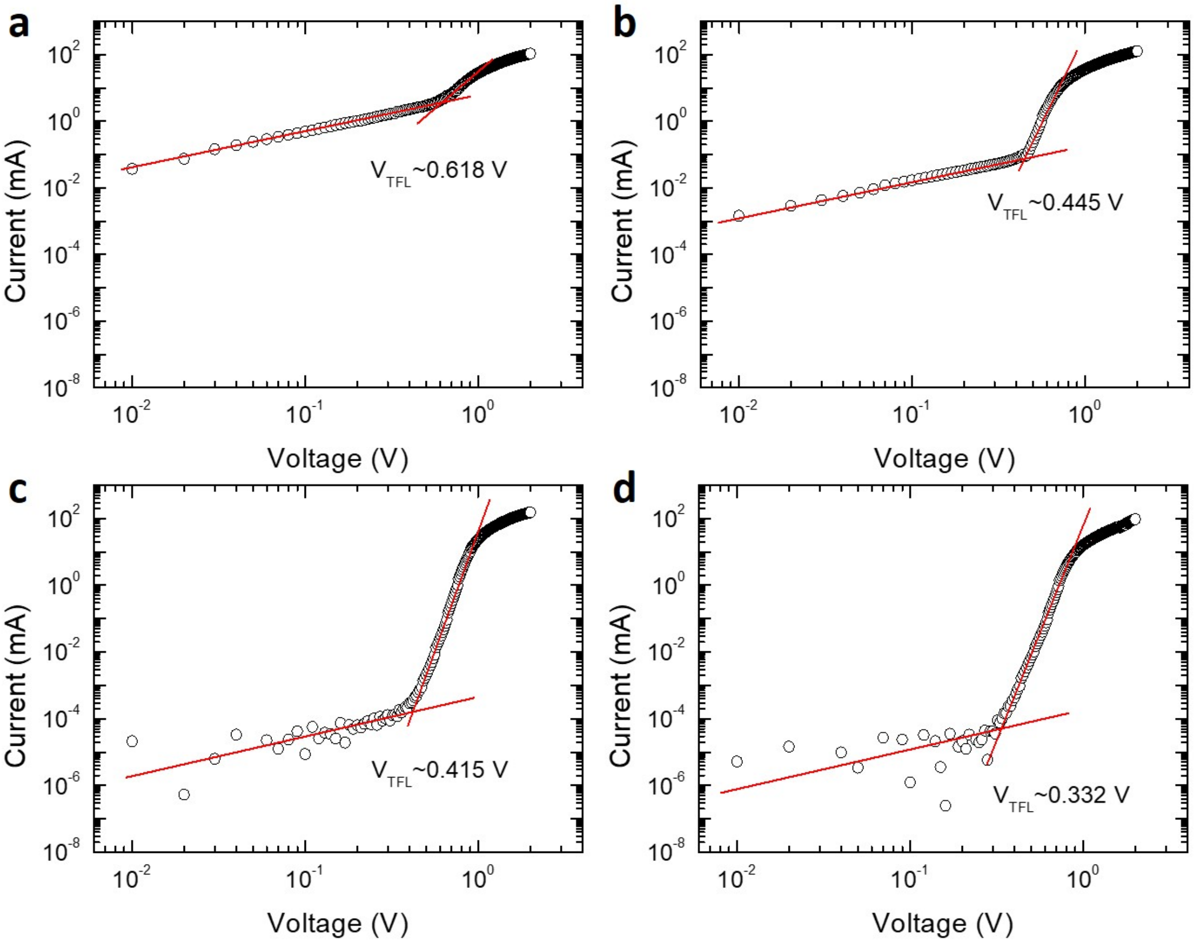

Supplementary Fig. 16 | Space charge limited current (SCLC) measurement of the $FAPbI_3$ films. a, control: bare $FAPbI_3$, **b**, 1P: $FAPbI_3$ with 1.67 mol% PEA_2PbI_4, **c**, 3P: $FAPbI_3$ with 3.33 mol% PEA_2PbI_4, **d**, 3F: $FAPbI_3$ with 3.33 mol% $FPEA_2PbI_4$. Trap filling voltages (V_{TFL}) for each graph was determined from the linear intercept of the red lines where the characteristic current-voltage relationship transfer from ohmic ($I \propto V$) to trap-filling ($I \propto V^{n>3}$) regime.

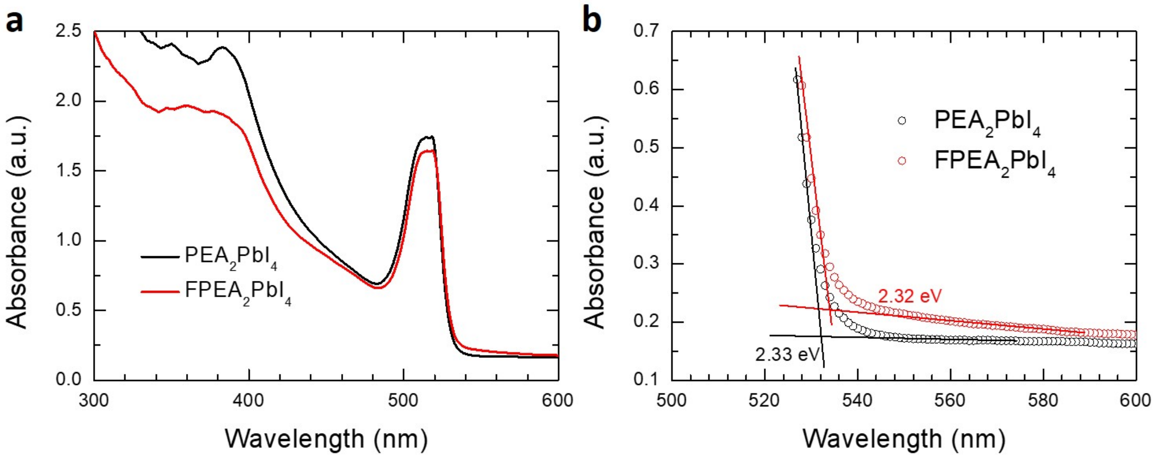

Supplementary Fig. 17 | Determination of bandgap of layered perovskites. a, UV-vis absorption spectra of PEA_2PbI_4 and $FPEA_2PbI_4$ films on a glass substrate. **b**, Linear interpolation of absorption onset region and calculated bandgaps.

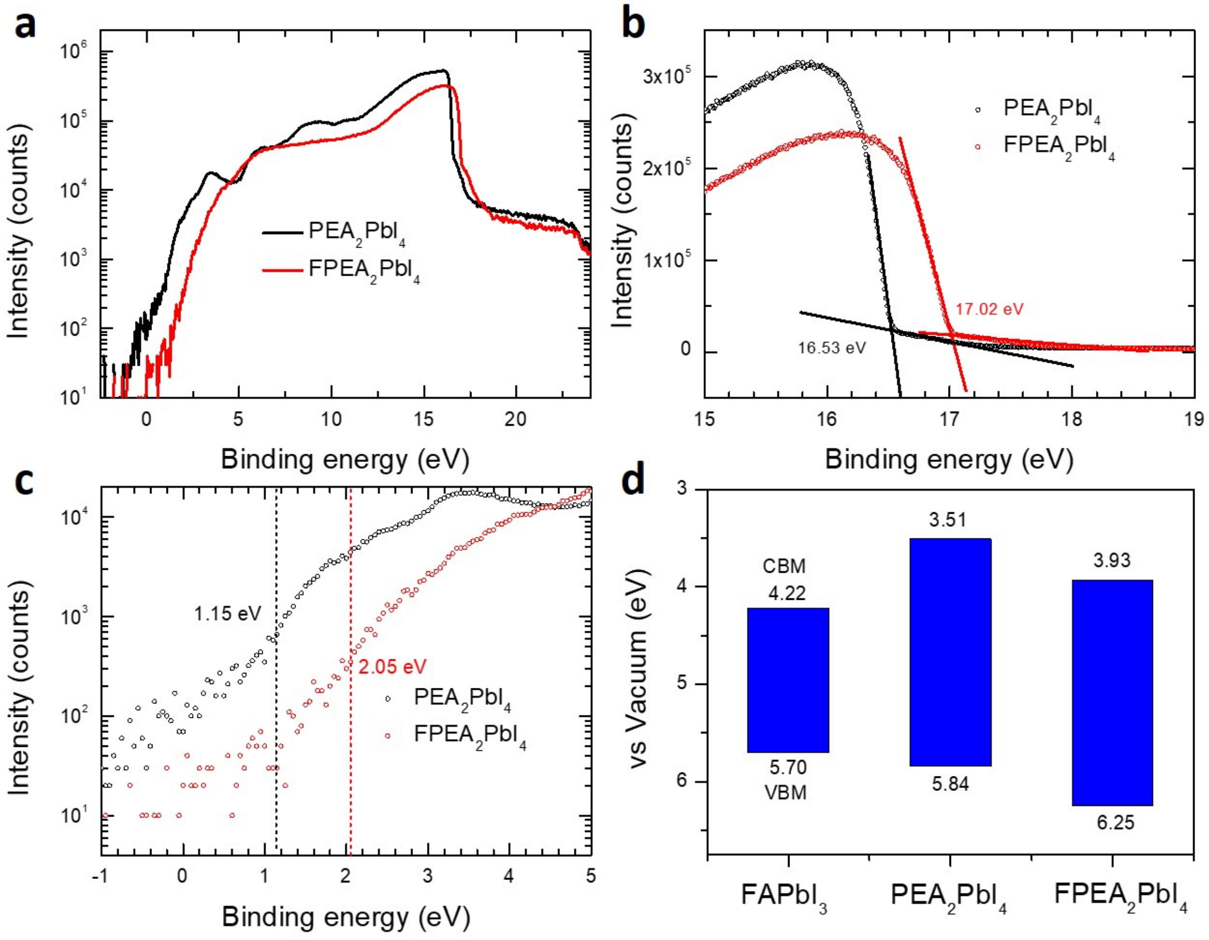

Supplementary Fig. 18 | Ultraviolet photoelectron spectroscopy (UPS) measurement of layered perovskites. **a**, UPS spectra of PEA_2PbI_4 and $FPEA_2PbI_4$ films on a ITO substrate. **b**, cutt-off and **c**, onset regions of the spectra. **d**, Calculated band structure of the α-$FAPbI_3$, PEA_2PbI_4 and $FPEA_2PbI_4$. CBM and VBM stand for conduction band minimum and valence band maximum, respectively.

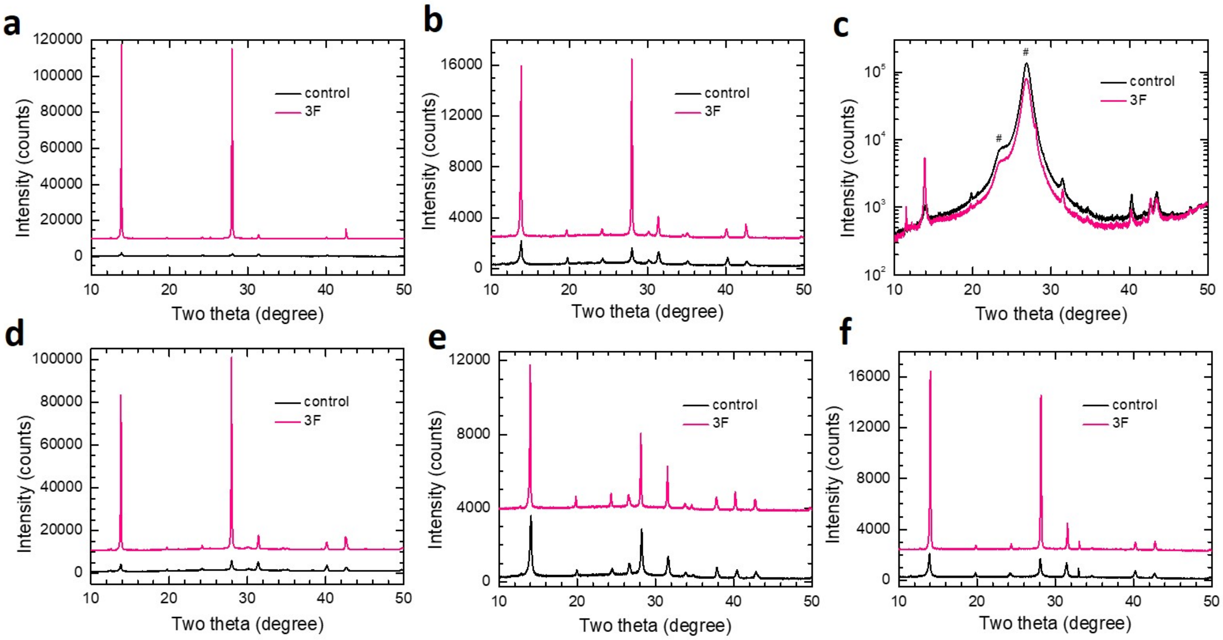

Supplementary Fig. 19 | X-ray diffraction (XRD) patterns on different substrates. XRD patterns of bare $FAPbI_3$ (control) and $FAPbI_3$ with addition of 3.33 mol% $FPEA_2PbI_4$ (3F). **a**, Glass, **b**, ITO, **c**, polyethylene naphthalate (PEN), **d**, SnO_2, **e**, fluorine doped tin oxide (FTO), and **f**, silicon wafer. The data obtained on PEN substrate is plotted in logarithmic scale to distinguish the signal from $FAPbI_3$ from huge substrate peaks (#).

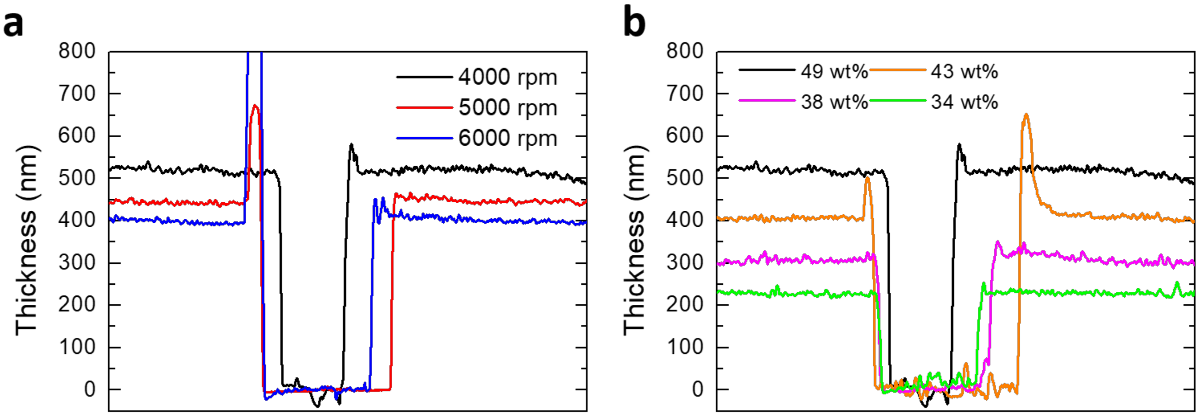

Supplementary Fig. 20 | Thickness control of NHE-$FAPbI_3$ films. Film thickness control by tuning the initial deposition of δ-$FAPbI_3$ as a function of **a,** different spin-coating deposition rate (rpm) and **b.** perovskite precursor concentration. The spin-coated δ-$FAPbI_3$ is then regularly annealed to trigger the NHE phase conversion process, and the thickness is measured after the NHE process.

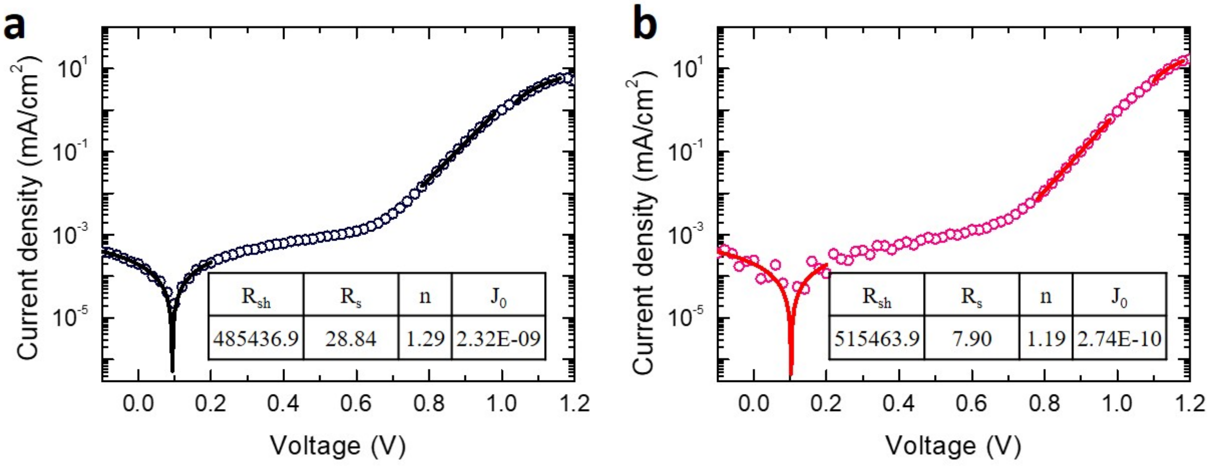

Supplementary Fig. 21 | Dark current measurement of solar cell devices. Dark current density-voltage (J-V) curves of solar cell devices based on a **a**, bare $FAPbI_3$ film (control) and **b**, $FAPbI_3$ film with 3.33 mol% $FPEA_2PbI_4$ (target). Open circles indicate measured data while solid lines indicate fitted curves. The fitted shun resistance (R_{sh}), series resistance (R_s), ideality factor (n) and saturation current (J_0) are presented in the tables.

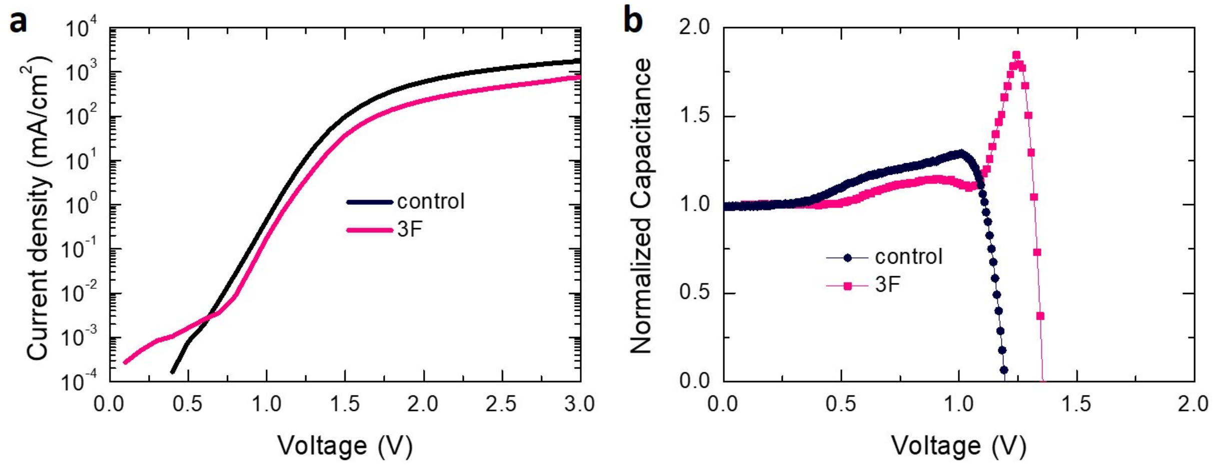

Supplementary Fig. 22 | Current density-voltage (J-V) and capacitance-voltage (C-V) curves of light emitting diode (LED) devices. a, J-V and **b**, C-V curves of LED devices based on bare $FAPbI_3$ film (control) and $FAPbI_3$ film with 3.33 mol% $FPEA_2PbI_4$ (3F).

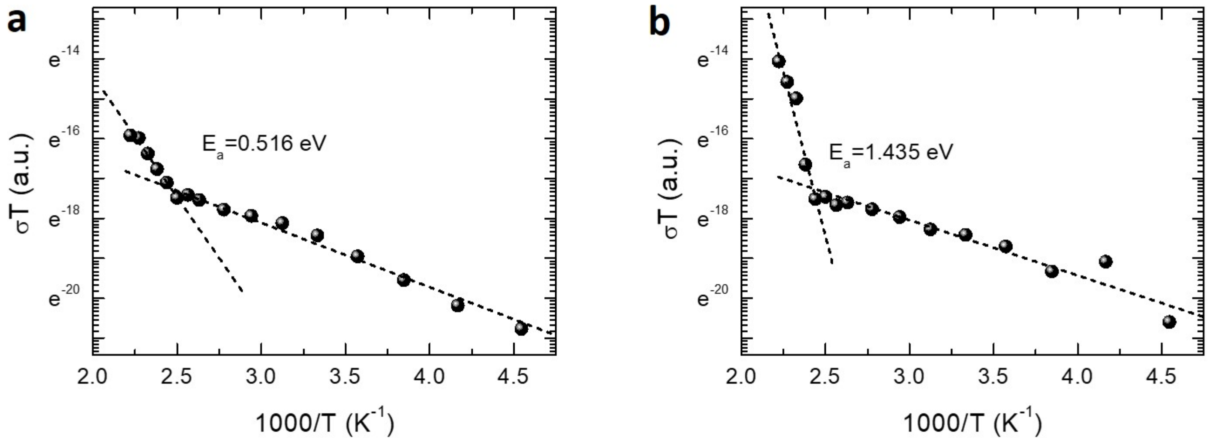

Supplementary Fig. 23 | Measurement of activation energies for ion migration. Temperature dependent current-voltage curves of Au/Perovskite (100 μm)/Au lateral devices based on **a**, bare $FAPbI_3$ (control) and **b**, $FAPbI_3$ film with 3.33 mol% $FPEA_2PbI_4$ (3F) films. The σT (σ is conductivity) and temperature relationship was plotted. Dashed lines indicate linear fitting of the data to calculate the activation energy for ion migration (E_a).

Supplementary Table 1| Extracted *k* values from the *in-situ* Grazing Incidence Wide Angle X-ray Scattering and X-ray diffraction monitoring of the phase transformation.

	***in-situ* GIWAXS**		**X-ray diffraction**	
	**n = 3** ***k***	**n = 4** ***k***	**n = 3** ***k***	**n = 4** ***k***
**control**	0.53818	0.5691	6.08	18.4
**1P**	7.04×10^{-3}	1.41×10^{-3}	6.45×10^{-2}	2.67×10^{-2}
**3P**	4.03×10^{-3}	7.01×10^{-4}	5.81×10^{-3}	1.17×10^{-3}
**3F**	3.68×10^{-4}	3.98×10^{-5}	3.15×10^{-3}	5.32×10^{-4}

Supplementary Table 2| Fitted peak positions and full-width-half-maximums ($\boldsymbol{\beta}_{\mathbf{total}}$) of the peaks extracted from the X-ray diffraction patterns of δ-$FAPbI_3$ films in Fig. 3a.

**Hexagonal $\boldsymbol{\delta}$-phase**				
**Bragg peak**	**R**	**1P**	**3P**	**3F**
(010)	2θ = 11.694°<br>β_{total} = 0.23712°	2θ = 11.676°<br>β_{total} = 0.25205°	2θ = 11.716°<br>β_{total} = 0.29268°	2θ = 11.698°<br>β_{total} = 0.19557°
(011)	2θ = 16.214°<br>β_{total} = 0.24919°	2θ = 16.179°<br>β_{total} = 0.25475°	2θ = 16.206°<br>β_{total} = 0.26732°	2θ = 16.185°<br>β_{total} = 0.20998°
(021)	2θ = 26.187°<br>β_{total} = 0.25294°	2θ = 26.166°<br>β_{total} = 0.27365°	2θ = 26.190°<br>β_{total} = 0.31578°	2θ = 26.180°<br>β_{total} = 0.23964°
(022)	2θ = 32.805°<br>β_{total} = 0.30102°	2θ = 32.779°<br>β_{total} = 0.32214°	2θ = 32.799°<br>β_{total} = 0.37408°	2θ = 32.783°<br>β_{total} = 0.28791°
(220)	2θ = 41.587°<br>β_{total} = 0.32823°	2θ = 41.563°<br>β_{total} = 0.36589°	2θ = 41.592°<br>β_{total} = 0.42321°	2θ = 41.588°<br>β_{total} = 0.34735°

Supplementary Table 3 | Fitted parameters for the data in Supplementary Fig. 10a-d. The crystallite size and strain were calculated based on the function described in the table.

δ-$FAPbI_3$ phase					
	**Function:** $\beta_{\text{total}} \cos\theta = 4\varepsilon \sin\theta + \frac{\lambda}{L}$			**Crystallite size (Å)**	**Strain (%)**
	**Slope**	**Intercept**	**R^2 value**		
**R**	0.00490	0.00354	0.869	435	0.123
**1P**	0.00648	0.00353	0.910	437	0.162
**3P**	0.00838	0.00379	0.869	406	0.209
**3F**	0.00887	0.00236	0.962	654	0.222

Supplementary Table 4 | Fitted parameters for the time-resolved photoluminescence decay profiles in Fig. 3j. The data were fitted with a bi-exponential decay model. Control: bare $FAPbI_3$, 1P: $FAPbI_3$ with 1.67 mol% PEA_2PbI_4, 3P: $FAPbI_3$ with 3.33 mol% PEA_2PbI_4, and 3F: $FAPbI_3$ with 3.33 mol% $FPEA_2PbI_4$.

	**Control**	**1P**	**3P**	**3F**
A_1 (%)	39.1	38.2	27.8	13.4
τ_1 (ns)	2.8	3.0	3.0	3.0
A_2 (%)	60.9	61.8	72.2	86.6
τ_2 (ns)	32.0	1212.6	1509.1	1862.1
Average τ_{avg} (ns)	20.6	750.8	1090.7	1613.6

Supplementary Table 5 | Summary of reported high-performance MA-free, or MA- and Br-free perovskite solar cells.

Composition	Device structure	E_g (eV)	V_{OC} (V)	J_{SC} (mA cm^{-2})	FF	PCE (%)	Ref
This work: NHE-$FAPbI_3$	ITO/SnO_2 /perovskite/spiro-OMeTAD/Au	1.48	1.10	24.93	0.79	21.6	-
Literature references for MA-free solar cells:							
$(Cs_{0.15}FA_{0.85})Pb(I_{0.9}Br_{0.1})_3$	ITO/PTAA/PFN-P2/perovskite/LiF/C60/BCP/Cu	1.58	1.11	23.19	0.80	20.7	4
$Cs_{0.15}FA_{0.85}Pb(I_{0.9}Br_{0.1})_3$	FTO/bl-TiO_2/perovskite/PbS/spiro-OMeTAD/Au	1.58	1.15	23.06	0.80	21.1	5
$BA_{0.05}(Cs_{0.17}FA_{0.83})_{0.95}Pb(I_{0.8}Br_{0.2})_3$	FTO/SnO_2/PCBM/perovskite/Spiro-OMeTAD/Ag	1.61	1.14	22.70	0.80	20.6	6
$(Cs_{0.17}FA_{0.83})Pb(I_{0.89}Br_{0.08}Cl_{0.03})_3$	FTO/bl-TiO_2/mp-TiO_2/SnO_2/perovskite/spiro-OMeTAD/Au	1.58	1.12	23.28	0.78	20.5	7
$(CsPbBr3)_{0.06}(FAPbI_3)_{0.94}$	FTO/bl-TiO_2/mp-TiO_2/perovskite/spiro-OMeTAD/Au	1.55	1.15	24.52	0.78	21.8	8
$Cs_{0.17}FA_{0.83}Pb(I_{0.8}Br_{0.2})_3$	ITO/PTAA/PFN-Br/perovskite/C_{60}/BCP/Cu	1.61	1.15	22.58	0.81	21.1	9
Literature references for MA-free, Br-free solar cells:							
$Rb_{0.05}FA_{0.95}PbI_3$	FTO/bl-TiO_2/mp-TiO_2/perovskite/spiro-OMeTAD/Au	1.53	1.07	23.93	0.67	17.2	10
$Cs_{0.1}FA_{0.9}PbI_3$	FTO/bl-TiO_2/ /perovskite/spiro-OMeTAD/Au	1.48	1.07	23.40	0.76	19.0	11
$Rb_{0.05}Cs_{0.10}FA_{0.85}PbI_3$	FTO/SnO_2/PCBM-PMMA/perovskite/Spiro-OMeTAD/Au	1.53	1.08	25.06	0.76	20.4	12
$Cs_{0.05}FA_{0.95}PbI_3$	ITO/PTAA/PFN-P2/perovskite/LiF/C60/BCP/Cu	1.50	1.05	25.10	0.75	19.8	13
$Cs_{0.02}FA_{0.98}PbI_3$	ITO/SnO_2 /perovskite/spiro-OMeTAD/Au	1.48	1.10	23.98	0.77	20.2	14
$[PEA_2PbI_4]_{0.167}[Cs_{0.02}FA_{0.98}PbI_3]_{0.9833}$	ITO/SnO_2 /perovskite/spiro-OMeTAD/Au	1.48	1.126	24.44	0.740	21.06	15

Literature references for MA-free, Br-free solar cells, with Cl (e.g. MACl)							
$FAPbI_3:(MDACl_2)_x$ (x = 3.8 mol%)	FTO/bl-TiO_2/mp-TiO_2/perovskite/passivation layer/spiro-OMeTAD/Au	1.47	1.14	26.50	0.82	24.7	[16]
$Cs_{0.2}FA_{0.8}PbI_3$-(Cl)	FTO/bl-TiO_2/mp-TiO_2/perovskite/spiro-OMeTAD/Au	1.56	1.10	24.10	0.78	20.6	[17]
$FAPbI_3$	FTO/SnO_2/Perovskite/Spiro-MeOTAD/Au	1.48	1.04	24.8	0.746	19.3	[18]

Supplementary References

1 Porter, D. A. & Easterling, K. E. *Phase transformations in metals and alloys (revised reprint)*. (CRC press, 2009).

2 Langford, J. I. & Wilson, A. Scherrer after sixty years: a survey and some new results in the determination of crystallite size. *J. Appl. Crystallogr.* **11**, 102-113 (1978).

3 Williamson, G. & Hall, W. X-ray line broadening from filed aluminium and wolfram. *Acta Metall.* **1**, 22-31 (1953).

4 Chen, Y. *et al.* Thermally stable methylammonium-free inverted perovskite solar cells with Zn^{2+} doped $CuGaO_2$ as efficient mesoporous hole-transporting layer. *Nano Energy* **61**, 148-157 (2019).

5 Chen, Y. *et al.* Interfacial Contact Passivation for Efficient and Stable Cesium-Formamidinium Double-Cation Lead Halide Perovskite Solar Cells. *Iscience* **23**, 100762 (2020).

6 Wang, Z. *et al.* Efficient ambient-air-stable solar cells with 2D–3D heterostructured butylammonium-caesium-formamidinium lead halide perovskites. *Nat. Energy* **2**, 17135 (2017).

7 Gao, X. X. *et al.* Stable and High-Efficiency Methylammonium-Free Perovskite Solar Cells. *Adv. Mater.* **32**, 1905502 (2020).

8 Xie, L. *et al.* Efficient and Stable Low-Bandgap Perovskite Solar Cells Enabled by a $CsPbBr_3$-Cluster Assisted Bottom-up Crystallization Approach. *J. Am. Chem. Soc.* **141**, 20537-20546 (2019).

9 Li, S. *et al.* Unravelling the Mechanism of Ionic Fullerene Passivation for Efficient and Stable Methylammonium-free Perovskite Solar Cells. *ACS Energy Lett.* (2020).

10 Park, Y. H. *et al.* Inorganic rubidium cation as an enhancer for photovoltaic performance and moisture stability of $HC(NH_2)_2PbI_3$ perovskite solar cells. *Advanced Functional Materials* **27**, 1605988 (2017).

11 Lee, J. W. *et al.* Formamidinium and cesium hybridization for photo-and moisture-stable

perovskite solar cell. *Adv. Energy Mater.* **5**, 1501310 (2015).
12 Turren-Cruz, S.-H., Hagfeldt, A. & Saliba, M. Methylammonium-free, high-performance, and stable perovskite solar cells on a planar architecture. *Science* **362**, 449-453 (2018).
13 Stolterfoht, M. *et al.* Visualization and suppression of interfacial recombination for high-efficiency large-area pin perovskite solar cells. *Nat. Energy* **3**, 847-854 (2018).
14 Lee, J.-W. *et al.* Tuning molecular interactions for highly reproducible and efficient formamidinium perovskite solar cells via adduct approach. *J. Am. Chem. Soc.* **140**, 6317-6324 (2018).
15 Lee, J.-W. *et al.* 2D perovskite stabilized phase-pure formamidinium perovskite solar cells. *Nat. Commun.* **9**, 1-10 (2018).
16 Min, H. *et al.* Efficient, stable solar cells by using inherent bandgap of α-phase formamidinium lead iodide. *Science* **366**, 749-753 (2019).
17 Prochowicz, D. *et al.* Engineering of perovskite materials based on formamidinium and cesium hybridization for high-efficiency solar cells. *Chem. Mater.* **31**, 1620-1627 (2019).
18 Yadavalli, S. K. *et al.* Mechanisms of Exceptional Grain Growth and Stability in Formamidinium Lead Triiodide Thin Films for Perovskite Solar Cells. *Acta Mater.* 193, 10-18 (2020).
