# Supplementary material for: Solid-phase hetero epitaxial growth of α-phase formamidinium perovskite
Source: Nat Commun. 2020 Nov 2;11:5514. doi: 10.1038/s41467-020-19237-3 (PMC7608657; doi:10.1038/s41467-020-19237-3)
Supplement: Supplementary file 3 — Reporting Summary [file 41467_2020_19237_MOESM3_ESM.pdf]

nature research

Corresponding author(s): Jin-Wook Lee, Yang Yang

Solar Cells Reporting Summary

Nature Research wishes to improve the reproducibility of the work that we publish. This form is intended for publication with all accepted papers reporting the characterization of photovoltaic devices and provides structure for consistency and transparency in reporting. Some list items might not apply to an individual manuscript, but all fields must be completed for clarity.

For further information on Nature Research policies, including our data availability policy, see Authors & Referees.

ü Experimental design

Please check: are the following details reported in the manuscript?

1. Dimensions

Item	Yes/No	Details
Area of the tested solar cells	[x] Yes [ ] No	Method section
Method used to determine the device area	[x] Yes [ ] No	Method section

2. Current-voltage characterization

Item	Yes/No	Details
Current density-voltage (J-V) plots in both forward and backward direction	[ ] Yes [x] No	Steady-state efficiency measurement was reported to ensure accurate efficiency measurement
Voltage scan conditions<br>*For instance: scan direction, speed, dwell times*	[x] Yes [ ] No	Method section
Test environment<br>*For instance: characterization temperature, in air or in glove box*	[x] Yes [ ] No	Method section
Protocol for preconditioning of the device before its characterization	[ ] Yes [x] No	No preconditioning was applied.
Stability of the J-V characteristic<br>*Verified with time evolution of the maximum power point or with the photocurrent at maximum power point; see ref. 7 for details.*	[x] Yes [ ] No	Steady-state efficiency measurement was reported.

3. Hysteresis or any other unusual behaviour

Item	Yes/No	Details
Description of the unusual behaviour observed during the characterization	[ ] Yes [x] No	no unusual behavior
Related experimental data	[ ] Yes [x] No	no unusual behavior

4. Efficiency

Item	Yes/No	Details
External quantum efficiency (EQE) or incident photons to current efficiency (IPCE)	[x] Yes [ ] No	Figure 4
A comparison between the integrated response under the standard reference spectrum and the response measure under the simulator	[x] Yes [ ] No	Figure 4
For tandem solar cells, the bias illumination and bias voltage used for each subcell	[ ] Yes [x] No	not applicable

5. Calibration

Item	Yes/No	Details
Light source and reference cell or sensor used for the characterization	[x] Yes [ ] No	Method section
Confirmation that the reference cell was calibrated and certified	[x] Yes [ ] No	Method section

Calculation of spectral mismatch between the reference cell and the devices under test

- [x] Yes
- [ ] No

It was reflected to the calibration processes.

6. Mask/aperture

Size of the mask/aperture used during testing

- [x] Yes
- [ ] No

Method section

Variation of the measured short-circuit current density with the mask/aperture area

- [ ] Yes
- [x] No

one kind of aperture was used

7. Performance certification

Identity of the independent certification laboratory that confirmed the photovoltaic performance

- [ ] Yes
- [x] No

It was not measured in the independent certification laboratory.

A copy of any certificate(s)
Provide in Supplementary Information

- [ ] Yes
- [x] No

It was not measured in the independent certification laboratory.

8. Statistics

Number of solar cells tested

- [ ] Yes
- [x] No

Only the best performing device was reported.

Statistical analysis of the device performance

- [ ] Yes
- [x] No

Only the best performing device was reported.

9. Long-term stability analysis

Type of analysis, bias conditions and environmental conditions
For instance: illumination type, temperature, atmosphere humidity, encapsulation method, preconditioning temperature

- [x] Yes
- [ ] No

Figure 4 and Method section
